# Relationships between Lifestyle, Living Environments, and Incidence of Hypertension in Japan (in Men): Based on Participant’s Data from the Nationwide Medical Check-Up

**DOI:** 10.1371/journal.pone.0165313

**Published:** 2016-10-27

**Authors:** Mayumi Oka, Mio Yamamoto, Kanae Mure, Tatsuya Takeshita, Mikio Arita

**Affiliations:** 1 School of Health and Nursing Science, Wakayama Medical University, Wakayama, Japan; 2 School of Public Health, Wakayama Medical University, Wakayama, Japan; Hamamatsu Ika Daigaku, JAPAN

## Abstract

This study aims to investigate factors that contribute to the differences in incidence of hypertension between different regions in Japan, by accounting for not only individual lifestyles, but also their living environments. The target participants of this survey were individuals who received medical treatment for hypertension, as well as hypertension patients who have not received any treatment. The objective variable for analysis was the incidence of hypertension as data aggregated per prefecture. We used data (in men) including obesity, salt intake, vegetable intake, habitual alcohol consumption, habitual smoking, and number of steps walked per day. The variables within living environment included number of rail stations, standard/light vehicle usage, and slope of habitable land. In addition, we analyzed data for the variables related to medical environment including, participation rate in medical check-ups and number of hospitals. We performed multiple stepwise regression analyses to elucidate the correlation of these variables by using hypertension incidence as the objective variable. Hypertension incidence showed a significant negative correlation with walking and medical check-ups, and a significant positive correlation with light-vehicle usage and slope. Between the number of steps and variables related to the living environment, number of rail stations showed a significant positive correlation, while, standard- and light-vehicle usage showed significant negative correlation. Moreover, with stepwise multiple regression analysis, walking showed the strongest effect. The differences in daily walking based on living environment were associated with the disparities in the hypertension incidence in Japan.

## Introduction

According to a report by the Ministry of Health, Labor and Welfare in Japan, approximately 60% and 40% of men and women, respectively, aged 40–74 years had hypertension (blood pressure >140/90 mmHg) [1]. The incidence of hypertension differs between the 47 prefectures in Japan, which vary in topography, climate, industrial structure, and population distribution.

There is a large volume of existing data regarding the causes of hypertension, with a high-salt diet, smoking, alcohol consumption, obesity, and lack of exercise considered especially strong risk factors [2–10]. Studies also indicate that low temperatures and large temperature variability are detrimental for blood pressure [11,12].

Japan has a national health insurance system, and all citizens aged 40–75 years who are covered by this insurance undergo a public medical check-up every year (henceforth referred to as “med check-up”) [13]. Tsushita et al. at the National Center for Geriatrics and Gerontology conducted analyses of 22.45 million of the 58.73 million people who underwent this med check-up in fiscal year 2010 and published the age-adjusted incidence of hypertension by prefecture [14]. The incidences of hypertension in Wakayama Prefecture, where the authors are located, were 28.3% for men (worst national rate) and 20.9% for women (second worst national rate); the total combined incidence for men and women was the highest in the nation.

Until 2011, when surveys per prefecture on the status of illnesses across the national population in Japan were performed, references were always made to the results of patient surveys carried out every three years by the Ministry of Health, Labor and Welfare (survey of approx. 13,000 people) [1]. These samples consisted only of people who had received some form of medical treatment, and the surveys did not adjust for the population age when aggregating total figures per prefecture.

On the other hand, the aforementioned survey analyzed a population sample of approx. 58 million people who received public medical check-up (total sample size of approx. 22 million people) [13], including people suffering from hypertension who received treatment for it, as well as those who did not. In addition, the survey was adjusted for the age of population when aggregating the total figures per prefecture.

The target populations of this survey were people who received medical treatment for hypertension, and also encompassed total figures including hypertension patients who did not receive any treatments. The analysis from this survey is based on statistical data per prefecture (with age adjustments), and it aims to investigate the factors that contribute to the differences in hypertension incidence across country, by taking into account individual lifestyles, as well as their living environments.

## Materials and Methods

The objective variable for the present survey analysis was hypertension incidence as data aggregated per prefecture; hence, the explanatory variables were also aggregated per prefecture or consisted of data capable of aggregation per prefecture.

First, we examined the data for analyzing the effect of resident lifestyles on hypertension incidence per region.

We used data from the National Health and Nutrition Survey by the Ministry of Health, Labor and Welfare [1]. This survey is conducted every year, randomly targeting approximately 300 districts in the country (about 21,000 people from approximately 6,000 households). The results are age-adjusted and aggregated by prefecture, and only the items that have confirmed statistical integrity are published.

We used the following data in the present study: obesity, daily salt intake (salt), daily vegetable intake (vegetable), presence or absence of habitual alcohol consumption (drinking), presence or absence of habitual smoking (smoking), and daily number of walking steps (walking). "Walking" was added to the analysis as an index of the amount of physical activity, which is known to affect hypertension incidence.

The survey reports included the proviso that there were few applicable women with respect to obesity, smoking, and drinking, resulting in a large coefficient of variation during the analysis. As a result, we decided to include only men in the analysis.

Next, we examined data related to people's living environments, which is considered as a factor that influences the amount of time spent walking. Previous studies have shown that the landscape of a living environment, availability of good footpaths, daily means of transportation, accessibility to shops, and altitude can influence the time that people spend for walking [15–19]. Based on this consideration, we used the number of rail stations per 100 km^2^ of habitable land ("stations"), standard-vehicle usage ("standard-vehicle"), light-vehicle usage ("light-vehicle"), and steepness of habitable land ("slope") as variables for which data aggregated per prefecture was available.

"Stations" is an index that represents the frequency of railroad usage as a mode of transport by the residents of a region. The authors calculated this index based on the locations of all train stations in the country [20] as published by the Ministry of Land, Infrastructure and Transport.

"Standard-vehicle" and "light-vehicle" are indices that represent the frequency of car usage as a mode of transport by the residents of a region, or their usage frequency per car model. "Standard" and "light" are classifications based on the body size of the car, with "light" vehicle being the smallest; hence, light vehicles are used often on narrow roads and across mountainous regions. For this, we referenced data published by the National Survey of Family Income and Expenditure [21].

"Slope" represents data denoting the steepness of a habitable land as a numerical value, with high values corresponding to very steep mountainous regions. This index was created by the authors in cooperation with a map company during a previous study [22–24]. Average values were calculated for each prefecture by extracting gradient of the land directly beneath approx. 20 million buildings such as private homes, public institutions among others, using data provided by the Geographical Survey Institute [25].

Finally, as indices of medical environment, we used the percentage of patients receiving public medical check-ups ("med-check"), along with the number of clinics and hospitals per 100 km^2^ of habitable land ("hospitals"). For the former, we referenced data published by the Ministry of Health, Labor and Welfare [26] and for the latter, we performed calculations based on locations of the clinics and hospitals as published by the said Ministry [27].

Table 1 shows the definitions of these variables.

**Table 1:** Definitions of the lifestyle and environmental variables used to test relationships with hypertension incidence.

Category	Variable Name	Units	Definition/Method of Measurement	Source
State of health	Hypertension Incidence by prefecture (Hypertension)	%	• Hypertension: systolic blood pressure ≥140 mmHg • Age adjusted	Tsushita K. Effective health policy development by local governments: guidance for the use of existing data
	Obesity Incidence (Obesity)	%	Obesity: body mass index ≥25.0 kg/m^2^ • Age adjusted	Ministry of Health, Labor, and Welfare "Citizen Nutrition & Health Survey" 2008–2012
Individual lifestyle	Salt Intake (Salt)	g/day	• Calculated from a food diary • Age adjusted	
	Vegetable Intake	g/day	• Measured using a scale • Age adjusted	
	Habitual Smoking (Smoking)	Yes or No	• Smoked tobacco daily or occasionally in the past month • Age adjusted	
	Habitual Alcohol Consumption (Drinking)	Yes or No	• Drank alcohol >3 days per week and drank more than approximately 180 mL on those days • Age adjusted	
	Daily Number of Walking Steps (walking)	Steps/day	• Wearing a pedometer from waking up to going to bed • Age adjusted	
Living environment	Number of Rail Stations per 100 km^2^ of Habitable Land (Hospitals)	Stations	Index of the accessibility of rail use	Author calculated using data from the Ministry of Land, Infrastructure, Transport and Tourism
	Standard-vehicle coverage rate (Standard-vehicle)	%	• Index of frequency of standard/light-vehicle use • The proportion of the number of standard/light-vehicles against the total number of registered vehicles per prefecture	National Survey of Family Income and Expenditure
	Light-vehicle coverage rate (Light-vehicle)	%		
	Slope of Habitable Land (Slope)	°	Index of steepness of the land where people live	Author calculated using data from the Ministry of Land, Infrastructure, Transport, and Tourism
Medical environment	Percentage of Patients Receiving Public Medical Check-up (Med check-up)	%	Index of people's awareness of disease prevention	Ministry of Health, Labor, and Welfare
	Number of Clinics + Hospitals per 100 km^2^ of Habitable Land (Hospitals)		Index of accessibility to medical institutions	Author calculated using data from the Ministry of Health, Labor, and Welfare

We performed correlation analyses on these variables using hypertension incidence per prefecture as the objective variable. In addition, we employed multiple stepwise regression analyses, in order to understand the factors that influence regional differences in hypertension incidence across the country.

SPSS Ver. 20 (IBM Corp, Armonk, NY, USA) was used for all analyses. The significance level was set at 5%.

## Results

The results of correlation analysis using hypertension incidence as the objective variable showed a significant negative correlation with walking (correlation coefficient -0.444) and med-check (-0.314), while a significant positive correlation with light-vehicle (0.437) and slope (0.321) were observed (Table 2).

**Table 2:** Correlations between lifestyle and living environment variables and the incidence of hypertension by prefecture in Japan.

		Obesity	Salt intake	Vegetable	Smoking	Drinking	Walking	Stations	Standard-vehicle	Light-vehicle	Slope	Med-check	Hospitals
Hypertension incidence (%)	Pearson correlation coefficient	0.258	-0.056	0.055	0.214	0.103	-0.044	-0.242	0.205	0.437	0.321	-0.314	-0.248

In addition, between number of steps and the variables related to living environment, stations (0.381) showed a significant positive correlation, while, standard-vehicle (-0.360) and light-vehicle usage (-0.537) showed significant negative correlations (Table 3).

**Table 3:** Correlations between the daily number of steps and environmental variables by prefecture.

	Stations	Standard-vehicle	Light-vehicle	Slope
Walking	0.381	-0.360	-0.537	-0.091

**Fig 1:**
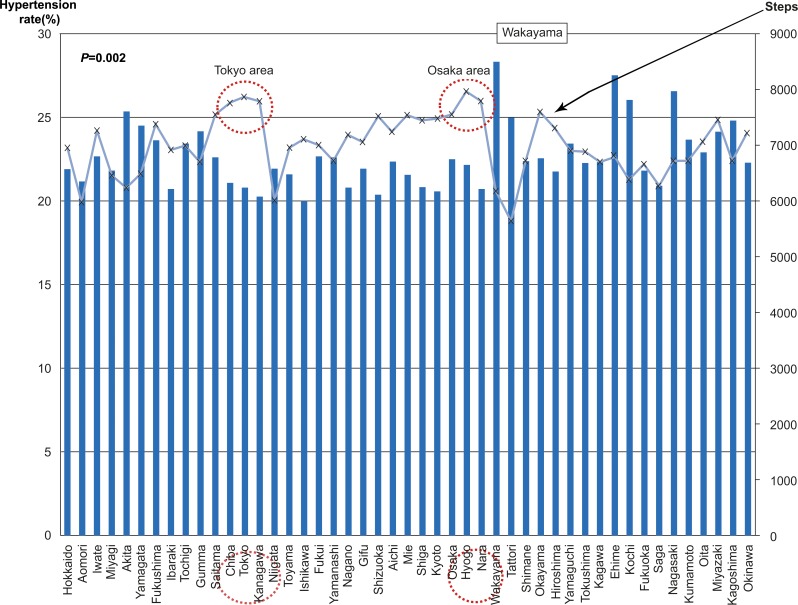
Relationship between hypertension incidence and daily number of steps by prefecture.

Fig 1 shows the relationship between hypertension incidence and number of steps within each prefecture. The number of steps was exceptionally high in two places: the capital city of Tokyo and its surroundings and the second-largest city of Osaka and its surroundings. Prefectures containing large metropolitan areas had a higher average number of steps.

The 47 Japanese prefectures on the x-axis are ordered from north to south from the northernmost prefecture of Hokkaido to the southernmost prefecture of Okinawa.

As a result of the stepwise multiple regression analysis with the incidence of hypertension as the outcome variable, walking and slope were selected, with walking having the strongest effect (R^2^ = 0.366, *P* < 0.001). The salt, vegetable, drinking, smoking, stations, standard-vehicle, light-vehicle, med check-up and hospitals were not significant (Table 4).

**Table 4:** Multivariate regression analysis with hypertension incidence as the dependent variable.

	Non-standardized coefficient		Standardized coefficient	*t* value	*P* value*	Collinearity statistics	
	B	Standard error	Beta			Tolerance	Variance inflation factor
Daily number of walking steps	0.001	0	0.426	3.527	0.001	0.989	1.011
Slope of habitable land	0.403	0.125	0.388	3.216	0.002	0.989	1.011

R^2^: 0.366

*P < 0.001

## Discussion

The novel component of this study is that the target population of analysis is comprised of both, people who have had medical treatments for hypertension, as in previous studies across the country, as well as the statistical data per prefecture that includes people suffering from hypertension but have not received medical treatment for this condition (with age adjustment).

This survey considered diverse aspects, including not only individual eating and fitness habits, but also their living environments. In this manner, the survey aimed to identify the factors that influence regional differences in hypertension incidence across the country.

A factor that significantly contributed to differences in the incidence of hypertension between the 47 prefectures was walking, with a lower walking associated with a higher incidence of hypertension. These findings support a number of previous studies indicating a relationship between physical activity and blood pressure [6,7,28].

There are a number of potential reasons for the regional differences in the number of steps. As a result of analysis, residents tended to take fewer steps in prefectures with low accessibility to railways; residents tend to rely on cars as their daily means of transportation in areas where the regional development of public transit is poor.

Also, as people's use of light-vehicle increases, walking decreases, and this effect was identified as stronger than the use of standard-vehicles. Wakayama Prefecture, which has the highest incidence of hypertension in Japan, also has the highest coverage proportion of light-vehicles in Japan, at 60.8%, which is considerably higher than the 6.2% in the capital area of Tokyo. As we mentioned, light vehicles are small and characterized by maneuverability in the narrow thoroughfares and agricultural roads frequently seen in Japan; this convenience might decrease the amount that residents walk. Compared to standard-vehicles, which tend to travel via parking lots, both at their departure and destination points, light-vehicles are able to travel door to door, creating a tendency to reduce people's number of steps even further. Hence, we can conclude that light-vehicle usage shows a stronger effect on walking.

Roughly 80% of Japan’s territory is mountainous. The percentage of the total land area that is habitable is 27.3%, which is significantly less than that in the UK (84.6%), Germany (66.7%), or France (72.5%) [29]. It is a cramped, precipitous terrain.

The particularly large number of steps by residents of metropolitan areas such as Tokyo and Osaka are likely due to a railway-centric lifestyle and lower use of car. In addition, the price of land is particularly high in urban areas due to Japan’s cramped and steep terrain, resulting in buildings with an ever-increasing number of floors both aboveground and below. Thus, residents navigate staircases frequently.

From the results of multiple regression analysis, the reasons for selecting walking as well as slope (which showed a significant negative correlation with the number of steps) is outlined below. In mountainous regions in Japan featuring highly steep slopes, temperatures tend to be low with many regions being covered in snow during the winter. A difference in temperature between indoors and outdoors causes the blood vessels to contract, suggesting an increased risk of developing hypertension.

No correlation was identified between hypertension incidence and the number of hospitals, which represents the level of medical facilities available.

In addition, based on the lack of an effect by the medical check-up participation rate on hypertension incidence, individual health awareness might have less of an impact than the living environment. In other words, living in environments that require more walking might suppress the incidence of hypertension irrespective of individual health awareness.

Not many analyses have been performed in Japan on the effect of living environments using hypertension as the objective variable. However, the results of our analysis actually concur with the results of a previous study, which indicates that longer the walking time when commuting, lower the risk of hypertension; as well as the a study which indicates that poor accessibility, leads to a higher risk of developing hypertension [30,31].

In our analysis, we used "slope" as one of the indices related to the land; however, a previous study [32] that used "elevation" instead, showed no significant correlation between hypertension and elevation. Although both elevation and slope are indices that represent mountainous regions, in our future studies we would like to investigate the hypothesis that even if elevation of a region is high, as long as there are no highly steep slopes, the risk of hypertension does not increase.

In addition, Oura et al. [33] state that a year-round subtropical climate could be the primary factor for Okinawa Prefecture's lower hypertension incidence compared to other prefectures. In the current study, we have not used any variables directly related to climate; however, a clear relationship has been identified whereby people living in mountainous regions with highly steep slopes experience higher hypertension incidence. Based on the fact that mountainous regions tend to feature lower temperatures, we can conclude that our findings are not in contradiction with the results of previous studies.

After all, salt, drinking and smoking as risk factors for hypertension might have been no significant effect in the present study because of the relatively small differences between the 47 prefectures.

This study focused on residential environment and did not consider the effect of social factors such as economic status and level of education. We aim to include these variables in future analyses.

## Conclusions

The differences in daily walking based on living environments are associated with the disparities in the hypertension incidence (in men) in Japan. Habitual walking is independent of personal health awareness, and characteristics of the living environment affect the development of walking habits. For disease prevention, in addition to interventions at the individual level, we must consider measures aimed at living environments.

## References

[1] Ministry of Health, Labor and Welfare. National Health and Nutrition Examination Survey Ministry of Health, Labor and Welfare 2006 Available: http://www.mhlw.go.jp/houdou/2008/04/h0430-2.html.

[2] Aburto NJ, Ziolkovska A, Hooper L, Elliott P, Cappuccio FP, Meerpohl JJ. Effect of lower sodium intake on health: systematic review and meta-analyses. BMJ. 2013;346: f1326 10.1136/bmj.f1326 23558163 PMC4816261

[3] Boeing H, Bechthold A, Bub A, Ellinger S, Haller D, Kroke A, et al Critical review: vegetables and fruit in the prevention of chronic diseases. European Journal of Nutrition 2012;51: 637–663. 10.1007/s00394-012-0380-y 22684631 PMC3419346

[4] He FJ, Li J, Macgregor GA. Effect of longer term modest salt reduction on blood pressure: Cochrane systematic review and meta-analysis of randomised trials. BMJ. 2013;346: f1325 10.1136/bmj.f1325 23558162

[5] Jonas DE, Garbutt JC, Amick HR, Brown JM, Brownley KA, Council CL, et al Behavioral counseling after screening for alcohol misuse in primary care: a systematic review and meta-analysis for the US Preventive Services Task Force. Ann Intern Med. 2012;157: 645–654. 10.7326/0003-4819-157-9-201211060-00544 23007881

[6] Newton RL Jr, Griffith DM, Kearney WB, Bennett GG. A systematic review of weight loss, physical activity and dietary interventions involving African American men. Obes Rev. 2014;15: 93–106. 10.1111/obr.12209 25196408

[7] Pate RR, Pratt M, Blair SN, Haskell WL, Macera CA, Bouchard C, et al Physical activity and public health. A recommendation from the Centers for Disease Control and Prevention and the American College of Sports Medicine. JAMA. 1995;273: 402–407. 7823386 10.1001/jama.273.5.402

[8] Rossi A, Dikareva A, Bacon SL, Daskalopoulou SS. The impact of physical activity on mortality in patients with high blood pressure: a systematic review. J Hypertens. 2012;30: 1277–1288. 10.1097/HJH.0b013e3283544669 22573122

[9] Taylor RS, Ashton KE, Moxham T, Hooper L, Ebrahim S. Reduced dietary salt for the prevention of cardiovascular disease: a meta-analysis of randomized controlled trials (Cochrane review). Am J Hypertens. 2011;24: 843–853. 10.1038/ajh.2011.115 21731062

[10] Tremblay MS, LeBlanc AG, Kho ME, Saunders TJ, Larouche R, Colley RC, et al Systematic review of sedentary behaviour and health indicators in school-aged children and youth. Int J Behav Nutr Phys Act. 2011;8: 98 10.1186/1479-5868-8-98 21936895 PMC3186735

[11] Mitchell R, Blane D, Bartley M, Elevated risk of high blood pressure: climate and the inverse housing law. Int J Epidemiol. 2002;31: 831–838. 12177031 10.1093/ije/31.4.831

[12] Wilmshurst P. Temperature and cardiovascular mortality. BMJ. 1994;309: 1029–1030. 7950726 10.1136/bmj.309.6961.1029 PMC2541551

[13] Ministry of Health, Labour and Welfare. The data about a specific medical checkup, specific health guidance Ministry of Health, Labour and Welfare 2010 Available: http://www.mhlw.go.jp/bunya/shakaihosho/iryouseido01/info02a-2.html.

[14] Tsushita K. Effective health policy development by local governments: guidance for the use of existing data Ministry of Health, Labour and Welfare Department 2013 Available: http://www.ahv.pref.aichi.jp/ct/other000001700/tebiki.4.pdf.

[15] Ishii K, Oka K, Inoue S, Shimotsu T. Association of built-environment and physical activity recommended for health promotion among Japanese adults. Japanese Journal of Health Education and Promotion. 2010;18(2): 115–125.

[16] Ishii K, Shibata A, Oka K, Inoue S, Shimomitsu T. Association of built-environment and active commuting among Japense adults. The Japanese Journal of Physical Fitness and Sports Medicine. 2010;59(2): 215–224.

[17] Christiansen LB, Madsen T, Schipperijn J, Ersboll AK, Troelsen J. Variations in active transport behavior among different neighborhoods and across adult lifestages. J Transp Health. 2014;1(4): 316–325. 10.1016/j.jth.2014.10.002 25506554 PMC4260423

[18] Chudyk AM, Winters M, Moniruzzaman M, Ashe MC, Gould JS, McKay H. Destinations matter: The association between where older adults live and their travel behavior. J Transp Health. 2015;2(1): 50–57. 10.1016/j.jth.2014.09.008 27104147 PMC4835227

[19] Durand CP, Tang X, Gabriel KP, Sener IN, Oluyomi AO, Knell G, et al The Association of Trip Distance With Walking To Reach Public Transit: Data from the California Household Travel Survey. J Transp Health. 2016;3(2): 154–160. 10.1016/j.jth.2015.08.007 27429905 PMC4941821

[20] Ministry of Land, Infrastructure, Transport and Tourism. Institution for Transport Policy Studies. Regional transportation annual report. 2014.

[21] Ministry of Internal Affairs and communications. National Survey of Family Income and Expenditure. 2014. Available at: http://www.stat.go.jp/data/zensho/2014/.

[22] Oka M, Fujita T, Yamauchi K. Analysis of geographical features in area with low suicide rates-standardized mortality ratio excerpted from Japanese municipal suicide statistics from 1973 to 2002. Journal of Health and Welfare Statistics. 2012;59(4): 1–9.

[23] Oka M. Social ecology and suicide: An analysis of topographic and climatic characteristics in areas with low and high suicide incidence. Psychologia. 2014;57(2): 65–81.

[24] Oka M, Kubota T, Tsubaki H, Yamauchi K. Analysis of impact of geographic characteristics on suicide rate and visualization of result with Geographic Information System. Psychiatry Clin Neurosci. 2015;69(6): 375–382. 10.1111/pcn.12254 25384900

[25] Geospatial Information Authority of Japan. Maps and geospacial information. Geographical Survey Institute 2016. Available: http://fgd.gsi.go.jp/download/menu.php.

[26] Ministry of Health, Labour and Welfare. Data of public medical check-up Ministry of Health, Labour and Welfare 2016 Available: http://www.mhlw.go.jp/bunya/shakaihosho/iryouseido01/info02a-2.html

[27] Ministry of Health, Labour and Welfare. Survey of medical facilities Ministry of Health, Labour and Welfare 2016 Available: http://www.mhlw.go.jp/toukei/list/79-1.html

[28] Jonas DE, Garbutt JC, Amick HR, Brown JM, Brownley KA, Council CL, et al Behavioral counseling after screening for alcohol misuse in primary care: a systematic review and meta-analysis for the US Preventive Services Task Force. Ann Intern Med. 2012;157: 645–654. 10.7326/0003-4819-157-9-201211060-00544 23007881

[29] Ministry of Land, Infrastructure, Transport and Tourism. The Land in Japan. Ministry of Land, Infrastructure and Transport 2006. Available: http://www.mlit.go.jp/common/000997376.pdf.

[30] Hayashi T, Tsumura K, Suematsu C, Okada K, Fujii S, Endo G. Walking to work and the risk for hypertension in men: the Osaka Health Survey. Ann Intern Med 1999;131(1): 21–26. 10391811 10.7326/0003-4819-131-1-199907060-00005

[31] Ohta Y, Kawano Y, Minami J, Iwashima Y, Hayashi S, Yoshihara F, et al Effects of daily walking on office, home and 24-h blood pressure in hypertensive patients. Clin Exp Hypertens 2015;37(5): 433–437. 10.3109/10641963.2015.1013115 25815710

[32] Hamano T, Kimura Y, Takeda M, Yamasaki M, Isomura M, Nabika T, et al Effect of environmental and lifestyle factors on hypertension: Shimane COHRE study. PloS one 2012;7(11): e49122 10.1371/journal.pone.0049122 23152860 PMC3494668

[33] Oura T, Mitsui G, Sakumoto S. Epidemiological study of hypertension in Okinawan people. Journal of health and human ecology 1990;56(2): 64–71.

